# Optimizing Hip Replacement Procedure in Cerebral Palsy-Related Spastic Hip Dysplasia: A Case Report

**DOI:** 10.7759/cureus.57584

**Published:** 2024-04-04

**Authors:** Androniki Drakou, Pavlos Altsitzioglou, Spyridon Sioutis, Anastasios G Roustemis, Dimitrios S Mastrokalos, Dimitrios Koulalis

**Affiliations:** 1 Department of Orthopaedic Surgery, Laiko University Hospital, Athens, GRC; 2 1st Department of Orthopaedic Surgery, Attikon University Hospital, Athens, GRC

**Keywords:** cerebral palsy, preoperative planning, hip navigation, total hip replacement, spinopelvic alignment

## Abstract

Cerebral palsy (CP) often results in severe hip issues, disrupting musculoskeletal development and mobility due to problems such as dislocations and contractures, aggravated by spasticity and heightened muscular tone. While total hip arthroplasty (THA) is required in CP patients, the procedure carries high risks due to concerns about dislocation and wear. This study explores a method of intraoperative navigation to precisely execute preoperative strategies for spinopelvic alignment and optimal cup placement. We discuss a case of a 22-year-old male CP patient with bilateral hip dislocations who experienced significant discomfort, impeding mobility and affecting his performance as a Paralympic rower. He underwent bilateral hip replacement surgeries, guided by preoperative gait analysis and imaging, with navigation aiding in accurate acetabular component placement and correction of excessive femoral anteversion using a modular stem. The patient achieved excellent stability in both standing and rowing postures.

Overall, computer navigation enhances complex hip repair by facilitating intraoperative data collection and precise execution of preoperative plans. This approach may extend the lifespan of prostheses, particularly by achieving precise acetabular component placement based on spinopelvic alignment principles, thereby offering significant benefits for CP patients undergoing THA.

## Introduction

Cerebral palsy (CP) is a condition characterized by a non-progressive brain disorder that affects the muscles and bones, often leading to the development of foot deformities, including equinus foot deformity; it may also cause hip issues. The incidence of this condition in industrialized nations is estimated to be between 1.5 and 2.5 children per 1000 live births [1]. The clinical hip symptoms are caused by spastic hemiplegia and include a broad range of conditions, ranging from hip subluxation to full dislocation, accompanied by painful deterioration [2]. Children with CP initially have normal hip development from birth until about 18 months of age. However, beyond this period, hip abnormalities arise due to muscular imbalance, and these issues tend to worsen with time. Without proper treatment, these conditions may progress to severe hip contractures or windswept deformity. By the age of five years, children notably experience instances of subluxation or dislocation, with the majority of cases being diagnosed as posterior dislocation, while anterior dislocation is rare and accounts for only 1.5% of cases [3].

In patients with CP who are nearing adulthood and experiencing painful arthritis, total hip arthroplasty (THA) is a potential treatment option; however, its use is a subject of controversy due to concerns of dislocation (up to 14%), aseptic loosening, infection, premature prosthesis failure, and other associated problems [4]. Proper placement of components is crucial for achieving a favorable result in THA. However, this task might be challenging in CP patients [5]. We describe the use of intraoperative computer navigation as a helpful tool to ensure accurate placement of the acetabular cup in a CP patient.

## Case presentation

The patient was a 22-year-old male who presented with a diagnosis of CP and subsequent spastic quadriplegia; he reported experiencing discomfort in both hips during ambulation and seated positions. Over the past five years, the patient had been actively engaged in training to pursue a career as a Paralympic rowing competitor. However, this pursuit had been impeded by chronic hip discomfort and persistent muscle contractions. The patient had sought intervention to address these issues, specifically requesting two hip joints to alleviate pain and offer an adequate range of motion, particularly while assuming the seated rowing posture.

Medical history

The patient had been born prematurely at 26 weeks of gestation, weighing 980 grams, and spent the first three weeks of his life in an incubator. His medical history had remained ambiguous until the age of 10 years. At that time, he had undergone a left femoral osteotomy to address his hip dislocation, but the procedure had been unsuccessful. Subsequently, at the age of 12 years, he had undergone a right femoral osteotomy, which had also proved to be ineffective. Simultaneously, he had undergone subtalar arthrodesis of the left foot to address the equinus deformity. Following these procedures, he had received sporadic botulinum injections exclusively in his adductor muscles and hip extensor muscles.

At presentation

**Figure 1:**
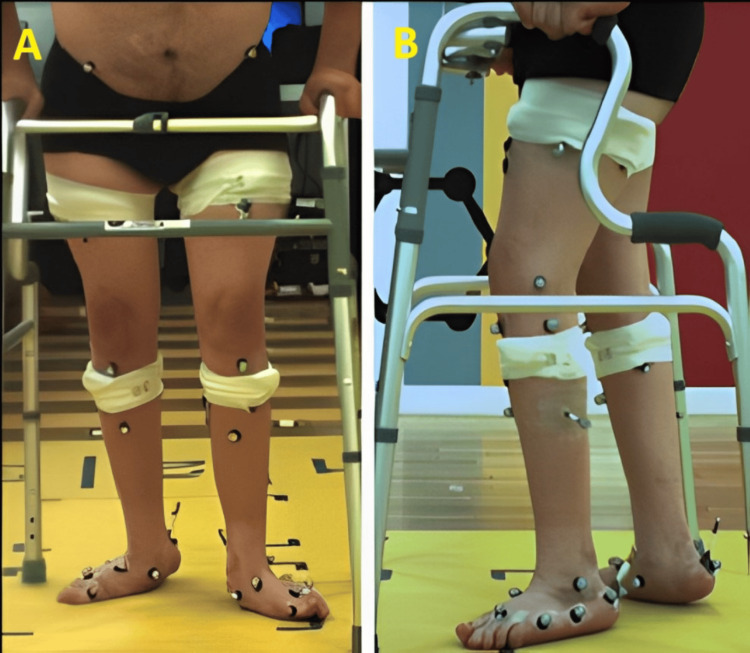
Images of the patient (A) anterior and (B) lateral position. Severe restriction in lower limb joint mobility in all directions was observed

He ambulated at a leisurely pace, taking small steps and moving his legs in a scissoring motion while relying on a cane for support. The mobility of his hips was severely restricted in all directions, and his adductor muscles were tight. His knees were flexed, and his ankles exhibited restricted mobility (Figure 1).

On examination

The hip flexors, abductors, adductors, knee flexors, and ankle dorsiflexors exhibited significant weakness. The hip and knee extensors required operation. Bilateral valgus flat foot was present, along with external tibial torsion on the right side, measuring more than 20 degrees.

Radiographs and CT scan

**Figure 2:**
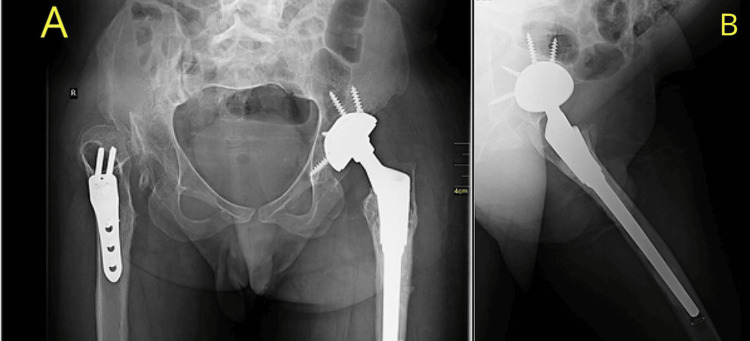
Imaging results A: When standing, the sacral slope was measured at 90 degrees, the pelvic tilt at 5 degrees, the pelvic incidence at 95 degrees, and the pelvic femoral angle at 155 degrees. B: When sitting the sacral slope was measured at 85 degrees, pelvic tilt at 5 degrees, pelvic incidence at 90 degrees, and pelvic femoral angle at 140 degrees

Bilateral high hip dislocations, bilateral acetabular dysplasia, and severe femoral anteversion were clearly seen on anteroposterior radiographs of the pelvis in a standing position. Both femurs included osteotomy fixation hardware (Figures 2A, 2B).

The CT scan of the pelvis revealed an acetabular anteversion of 23 degrees on the right side and 19 degrees on the left side. Additionally, the anterior column was seen to be inadequate.

Preoperative planning and surgery

Given the patient's neurological condition and muscular imbalance, it became evident that a restricted prosthesis with dual mobility articulation was the appropriate choice. However, we deemed this approach insufficient due to potential postoperative concerns about impingement and early wear. Hence, we conducted a comprehensive preoperative planning process, which involved measuring the sagittal spinopelvic dimensions. This enabled us to determine the ideal placement of the acetabular cup, ensuring the proper opening of the acetabulum in a sitting-rowing posture while minimizing the risk of dislocation or impingement. The patient's spinopelvic values indicated minor changes during the transition from standing to sitting positions, suggesting a stable pelvis with limited movement (standing SS:90, PI:95; sitting SS:85, PI:90) [6]. To maintain the stability of the prosthesis when sitting, we opted to increase acetabular anteversion, as it is counteracted by hip joint movement. Additionally, the forward pelvic angle exhibited limited transitions from standing to sitting positions, corresponding with the presence of biologically fused-like hips. To address both the spinopelvic and hip contractures, we utilized an acetabular component with high inclination and anteversion angles to mechanically alleviate the restriction. Consequently, cup implantation was performed with an abduction angle of 50 degrees and an anteversion of 25 degrees, utilizing the Naviswiss intraoperative hip navigation system (Naviswiss AG, Brugg, Switzerland).

To correct the excessive forward rotation of the thigh bone, we employed a modular prosthesis with adjustable forward rotation. During the surgery, the prosthesis was aligned with the trial acetabular cup, set at a retroversion angle of 20 degrees. Given the severe deficiency of the native acetabulum in both the front and back, the acetabular shell was placed at an elevated hip center, secured using a press-fit method, and reinforced with four screws around its circumference, with at least one screw in the ischium. The femoral stem was cemented due to severe osteoporosis resulting from lack of usage. The psoas tendon was released from the lesser trochanter using an open method, while the adductors were released from the pubis using a closed technique. Post-surgery, the patient mobilized the day after, refraining from bearing weight on the affected area, engaging instead in isometric exercises targeting the quadriceps and abductor muscles. Range of motion exercises for the hip allowed flexion of up to 100 degrees and complete extension of the operated hip.

## Discussion

Despite the prevalence of symptomatic hip arthritis in people with CP, surgeons have traditionally been hesitant to perform complete hip arthroplasties in this population due to apprehensions about dislocation and premature prosthesis malfunction. The objective of this pilot case study was to investigate whether the precise placement of the acetabular component using computer navigation could prevent the occurrence of postoperative outliers such as impingement, early wear, and loosening. Additionally, it aimed to determine if this technique would enable the patient to participate in their chosen sport, i.e., rowing.

Positioning components in individuals with CP is challenging due to the presence of hip flexion and adduction contractures, subluxation or dislocation, coxa valga, and increased femoral anteversion [5]. CP patients often exhibit spinopelvic imbalance, which is compensated for in the sagittal plane by knee flexion, hip flexion, pelvic anteversion, and forward inclination of the spine. This would manifest as elevated SS (sensory sensitivity) and reduced PFA (processing speed), as shown by the values of SS (85-90) and PFA (140-155) in this particular situation. The "safe zone" for the acetabular cup, as defined by Lewinnek et al. [7], aims to reduce the likelihood of dislocation following primary total hip replacement (THR). However, patients with a sagittal spinal deformity (SSD) and muscle imbalance are not afforded the same protection by the "safe zone". Utilizing dual mobility acetabular prostheses is necessary to treat the existing issues. However, it is also important to avoid the disruptive forces that impact the dual mobility articulation, as these pressures might cause premature loosening of the prosthesis.

Before commencing the procedure, it is essential to engage in meticulous preparation. Typically, the whole procedure is conducted using an anteroposterior pelvic X-ray. Special lateral views are conducted to assess spinopelvic alignment anomalies since they enable the measurement of more advanced aspects of pelvic alignment. This visualization should depict the anatomical region spanning from the L1 vertebra to the proximal femur, including the pelvis [8]. Spinal imbalance may arise from two structural alterations: either stiffness or hypermobility [6]. The imbalance in individuals with CP is caused by rigidity in both the spinopelvic junction and the hip joints. This is apparent from the measurements of sacral tilt, pelvic incidence, and pelvic-femoral angle, which show little variation while transitioning from a standing to a sitting posture (in our case, the results only alter by up to 5 degrees). To achieve a mechanically open acetabular component for sitting and to enhance stability and reduce pressures at the back of the prosthesis when sitting, hips with a fixed pelvis need a significant inclination and anteversion [6]. Intraoperative computer navigation devices enable precise placement of the acetabular cup.

Typically, limited movement in the spine and pelvis is balanced by more movement in the hip joints. However, if the hip joint has less covering in the lower part, there is a higher chance of the hip dislocating backward while sitting [9,10]. Dual mobility cups have the potential to experience eccentric loading from dislocating pressures while in the sitting position, and also in the rowing position, despite their reduced risk of dislocation. Pelvic tilt is often assessed by surgeons just before acetabular reaming when the patient is in a supine posture. The patient will not maintain this posture continuously, and the positioning of the acetabulum should be adjusted to accommodate not only this position (which deviates somewhat from the standing position) but also a sitting position [9].

Regrettably, evaluating the functional status of patients with CP after THA is challenging due to the inability to standardize their status according to the existing total hip rating systems [11]. CP causes stiffness, which restricts the range of motion and limits the distance that patients can walk. As a result, patients often need to use gait aids or can only walk short distances. The durability of the dual mobility prosthesis may be used as a measure of the correct placement of the implant. In this case, it is advisable to monitor the patient for the long term.

## Conclusions

We propose that utilizing computer navigation to precisely position the acetabular component during total hip replacement surgery in patients with CP can enhance the procedure by optimizing spinopelvic alignment. This alignment reduces the strain on the posterior aspect of the prosthesis while sitting, potentially leading to increased longevity of the implant. Typically, cup placement for patients with CP involves selecting greater abduction and anteversion angles to promote comfortable sitting. Further studies with longer follow-up periods have to be conducted to gain deeper insights into this topic.
